# A non-lethal method for the sampling of the gut microbiota in *Betta splendens*

**DOI:** 10.3389/fvets.2026.1702535

**Published:** 2026-02-05

**Authors:** Ka Ieng Pun, Vasana Jinatham, Muhammad Bashir Saidu, Manuel Sapage, Siam Popluechai, André Antunes, Kritsakorn Saninjuk, David Gonçalves

**Affiliations:** 1 Institute of Science and Environment, University of Saint Joseph, Macao, China; 2 School of Science, Mae Fah Luang University, Mueang Chiang Rai, Thailand; 3 Gut Microbiome Research Group, Mae Fah Luang University, Mueang Chiang Rai, Thailand; 4 William James Center for Research, ISPA—Instituto Universitário, Lisbon, Portugal

**Keywords:** 16S rRNA gene sequencing, aggression, fish microbiota, sampling procedure, Siamese fighting fish, swab collection method

## Abstract

The gut microbiota is known to modulate brain function and behavior but the underlying mechanisms are still poorly understood. In particular, research on the impact of gut microbiota in aggressive behavior is scarce. The Siamese fighting fish (*Betta splendens*) has been increasingly used for studies of aggression but methodologies for gut microbiota-aggression studies are lacking for the species. Here, we compared the gut microbial diversity in *B. splendens* using a conventional lethal gut sampling technique and a non-lethal mouth swabbing method. Bacterial communities were profiled by 16S rRNA gene sequencing targeting the V3–V4 region. Alpha diversity analyses, including observed amplicon sequence variants (ASV), Shannon diversity index, Chao1 richness estimator, and Faith’s phylogenetic diversity, revealed no statistically significant differences between sampling methods when compared using Kruskal–Wallis and Wilcoxon rank-sum tests. Beta diversity analyses using Bray–Curtis dissimilarity index through PERMANOVA also held comparative results, although phylogenetically informed metrics, such as weighted UniFrac and generalized UniFrac, revealed significant compositional divergence between the two sampling methods, with the swab collection method holding generally higher values. The swab method identified most of the microbial groups also identified by lethal gut sampling, plus some additional taxa that are potentially only present in the upper gut. Taken together, our findings support the use of non-lethal mouth swabbing as a viable and ethically preferred alternative to traditional sampling of the intestine for characterizing the gut microbiota of *B. splendens*. This technique, first applied in a small fish, may be particularly valuable in longitudinal studies to assess changes in gut microbiota in relation to aggression in *B. splendens*, enabling repeated sampling over time to investigate how microbial community dynamics correlate with the development, expression, or modulation of aggressive behavior.

## 1 Introduction

The gut microbiota–brain axis, a bidirectional communication network involving neural, hormonal, immune, and metabolic pathways (1), plays a key role in host physiology, extending beyond digestion and immunity to influence brain function and behavior. Microbial signals can modulate stress reactivity, emotional regulation, and social behavior, while the brain, in turn, can affect gut function and microbial composition (2, 3). Although its behavioral relevance is now unquestionable, the exact mechanisms of this communication remain poorly elucidated.

Fish are increasingly recognized as powerful models for studying the gut microbiota–brain axis due to their wide ecological diversity, neurochemical homologies with mammals, and suitability for high-throughput, controlled experimentation. However, this potential cannot be fully realized because investigation of fish gut microbiota predominantly employs lethal sampling methods (4–6), thus missing the opportunity to be used in rare or threatened populations and species and in gut microbiota dynamics during longitudinal studies (7). Gut microbiota studies using non-lethal sampling techniques is common within some vertebrate taxa (8–11) but remains underused in fish.

To transition from lethal to non-lethal methods in gut–microbiota sample collection, comparative assessments between microbial profiles are needed to identify potential biases between different sampling methods [e.g., (10–12)]. Sampling the gut microbiota with a mouth swab, a non-lethal method, reduces harm to animals, meets ethical research standards, and allows the same individual to be sampled multiple times without causing permanent damage. This makes long-term monitoring possible, facilitating the study of microbial communities changing over time.

Comparative assessments of alternative gut microbiota sampling methods in fish are scarce. In zebrafish (*Danio rerio*), Thormar et al. (13) evaluated four distinct sampling methodologies for collecting gut microbiota samples, each with their own advantages: (1) intestinal tissue sampling, which can sample intact bacterial flora and it is conducive to gene integration; (2) the analysis of intestinal contents, which has high recovery rate; (3) extrusion sampling, which can capture high diversity and it is conducive to functional research; and (4) fecal samples, which is easy to collect and suitable for long-term research. In another study, Ruiz et al. (14) compared gut microbiota samples obtained from scraping the mucosa and sampling the posterior intestine of the rainbow trout with a mouth swab and found quantitative but no qualitative differences between the bacterial composition of the samples.

The Siamese fighting fish, *Betta splendens*, is a highly popular and morphologically diverse ornamental fish (15), domesticated for hundreds of years and known for the high aggressiveness of males. As such, this species has been used in many ethological studies focusing on aggression (16) and could become a good candidate model for the study of the gut microbiota–brain–behavior axis, especially related to aggressive behavior. However, to date, there was only one study describing *B. splendens* gut microbiota, and this study applied lethal methods for gut microbiota sampling and did not focus on aggression (17).

In this study, we conducted a comparative analysis of gut microbial diversity in *B. splendens* using a conventional intestinal sampling method and a non-lethal swabbing technique, aiming to validate the latter as a non-lethal technique for the study of *B. splendens* gut microbiota. This is, to our knowledge, the second study applying the swabbing technique in fish [other than Ruiz et al. (14)], and the first study applying this method in small fish. If both methods are comparable in terms of sampled microbiota, which we expect, the swabbing technique could open new research opportunities for the study of the gut microbiota in *B. splendens*, such as in the context of the brain–gut microbiota interaction. As a broader application, validation of the swab technique in small fish will also facilitate its use in other species that are widely used as models in biomedical research or in ecotoxicology (e.g., zebrafish, *D. rerio*) (18, 19).

## 2 Materials and methods

### 2.1 Ethics statement

All procedures were conducted in accordance with animal ethics protocols approved by the Division of Animal Control and Inspection of the Civic and Municipal Affairs Bureau of Macao (AL017/DICV/SIS/2016).

### 2.2 Fish sampled

A total of 19 adult female *B. splendens* from a line selected for aggression maintained for 10 generations under laboratory conditions were used in this study (swab: *n* = 10; intestinal sampling: *n* = 9; but two samples from the intestinal sampling group were excluded, see below). Fish were maintained in a mixed-sex group tank (100 W × 50D × 30H cm, about 70 fish), with ceramic shelters and aquatic plants and external filtration. Reverse osmosis water with a salinity of 250 ppm, at a temperature of 24–26 °C, and a photoperiod of 12L:12D, from 8 a.m. to 8 p.m., was applied. Fish were fed in the morning with dry food (Atison’s Betta Pro, about 3.5–4 mg per fish), and in the afternoon with live artemia (about 2 mL of concentrated *Artemia salina*). Microbial samples were collected around noon, 20 h after feeding the live artemia the day before. Because the intestinal evacuation time for a small meal is estimated to be 4 to 6 h for similar-sized tropical carnivorous fish (20), the intestinal content was considered to be negligible at the time of sampling.

### 2.3 Sample collection

For the non-lethal mouth swabbing technique (swab sample collection), 10 fish were immersed in buffered tricaine methane sulfonate (400 mg/L of MS-222) until fully anesthetized. Length and weight of the fish were recorded. The swab samples were collected following the protocol of Ruiz et al. (14) with some modifications. The digestive tract of *B. splendens* is gastric, consisting of a stomach and a short, tubular intestine that lacks pyloric caeca, measuring approximately 0.5 to 0.8 times the standard body length of the fish, forming a single loop. Sampling targeted the middle of the intestine, which was selected for its consistent accessibility and being the part of the intestine less influenced by external (anterior or posterior) microbiota contamination. Additionally, in fish studies, is usual to target the whole intestine for microbiota analysis (21, 22), so in small fish like *B. splandens*, we considered that targeting the middle of the intestine can result in sampling the most representative microbiota communities in the intestine in a consistent manner. We used fish from our lab that naturally died to determine the depth necessary for the swab to reach this area of the digestive system. A swab was inserted into these fish with their gut open to visually determine the necessary depth for sampling, which was established to be around 1.4–1.8 cm for adult fish, like the ones used in this study. A sterile nylon swab (Ø = 1 mm, model 112698, LP Company, Italy) was quickly introduced through the fish’s mouth to a depth of 1.4–1.8 cm and rotated three times in a clockwise or counterclockwise manner. The swab was withdrawn, and its tip was cut with a sterile blade to preclude contamination. Due to the swab passing through anterior parts of the fish, some microbial communities from these regions can also appear in the samples, although the sampling method was designed to maximize the sampling of gut microbiota in the middle of the intestine and minimize the sampling in the mouth and anterior areas of the digestive tract. The tip was then stored in a sterile tube, and the fish returned to the water for recovery. For conventional lethal gut sampling technique (gut sample collection), nine fish were immersed in buffered tricaine methane sulfonate (400 mg/L of MS-222), until euthanized. The intestines were collected following the protocol of Wu et al. (23), with some modifications. Briefly, each fish was dissected and the intestine, from the anus backward, was aseptically extracted with disinfected scissors and placed in a sterile tube. Both swab and gut samples from each specimen were individually stored at −20 °C until further DNA extraction.

### 2.4 DNA extraction

DNA was extracted using the DNeasy Blood and Tissue Kit (Lot number 178015356, QIAGEN), following the manufacturer’s protocols. For samples collected from oral swabs, the complete cotton swab was incorporated into the lysis and homogenization step. For intestinal samples, the entire gastrointestinal tract, including the intestinal wall and the luminal content, was used for DNA extraction. Genomic DNA concentration was measured using Qubit and DNA stored at −20 °C until use. Following DNA extraction and 16S rRNA gene sequencing, all samples underwent a quality control step. Two samples from the intestinal group had few reads or exhibited poor sequencing quality and were excluded from subsequent analysis. Given the usage of the entire gastrointestinal tract for DNA extraction, the microbial community analyzed from these dissection-derived samples represents the total community associated with the intestinal tissue, encompassing both mucosa-associated and luminal microorganisms.

### 2.5 16S rRNA gene amplicon sequencing

Genomic DNA was processed for 16S rRNA gene amplification using 30 ng template DNA. The V3–V4 hypervariable region was targeted using the fusion primers containing Illumina adapters and sample specific barcodes. PCR products were purified with Agencourt AMPure XP Magnetic beads to eliminate primer-dimers and other small fragment contaminations. The purified DNA amplicons were subsequently ligated with a sequencing adapter. Fragment size and concentration were determined using the Agilent 2100 Bioanalyzer, confirming amplicon sizes ranging from 550 to 650 bp. Libraries were normalized to 4 nM concentration prior to sequencing. High-throughput sequencing was performed using MGI’s DNBSEQ^™^ technology, a DNA Nanoball-based paired-end sequencing system (PE300), ensuring high-throughput and high-accuracy sequencing.

### 2.6 Bioinformatics and statistical analysis

Raw sequencing data were processed using QIIME2 (version 2024.5.0). Briefly, adapter and primer sequences were removed using the Cutadapt plugin (24). Quality filtering, denoising, merging, and chimera removal were performed using the DADA2 plugin (25), generating high-quality amplicon sequence variants (ASVs) for downstream analysis. The ASVs were then aligned using MAFFT (26) and used to construct a phylogenetic tree with FastTree (27). Taxonomic assignment was performed using classify-sklearn naive Bayes taxonomy classifier (28) against the Silva database (silva-138.2-ssu-nr99, Released July 11, 2024). Data visualization was performed using the ggplot2 and pheatmap R packages.

Alpha diversity indices, including Observed ASVs, Chao1 richness, Shannon, and PD whole trees, were computed and visualized using the phyloseq R package (29). Pairwise comparison of alpha diversity between sampling methods was assessed using Kruskal–Wallis sum-rank test (alpha level determined at *p* < 0.05). Beta diversity was conducted using multiple dissimilarity matrices, including Bray–Curtis dissimilarity, unweighted UniFrac, weighted UniFrac and GUniFrac and visualized through principal coordinate analysis (PCoA) using the phyloseq R package. permutational multivariate analysis of variance (PERMANOVA) was calculated to assess significant differences in community composition between sampling methods (*p* < 0.05). Additionally, linear discriminant analysis effect size (LEfSe) (30), which employs a linear discriminant analysis (LDA), was used to identify differentially abundant taxa between gut and swab samples. We used a threshold score of 3.0 for the LDA, with a higher score indicating a more significant result. Kruskal–Wallis sum-rank test was applied to determine statistically significant bacterial biomarkers across sample groups. Data visualization was performed using the ggplot2 and pheatmap R packages. Functional potential of the microbial communities was predicted using PICRUSt2 (31). The predicted functional profiles were categorized according to KEGG Orthology (KO) terms and EC (Enzyme Commission) numbers. Differentially abundant taxa and inferred pathways resulting from the predicted functional abundance tables were visualized in heatmaps and principal component analysis (PCA) using the pheatmap and ggpicrust2 R packages, respectively.

## 3 Results

Data from a total of 10 female fish from the swab sampling and a total of seven female fish from the intestine sampling were used in this analysis. Fish body weight from both groups were confirmed not to be significantly different (independent *t*-test, *t* = −1.062, df = 15, *p* = 0.31).

**Figure 1:**
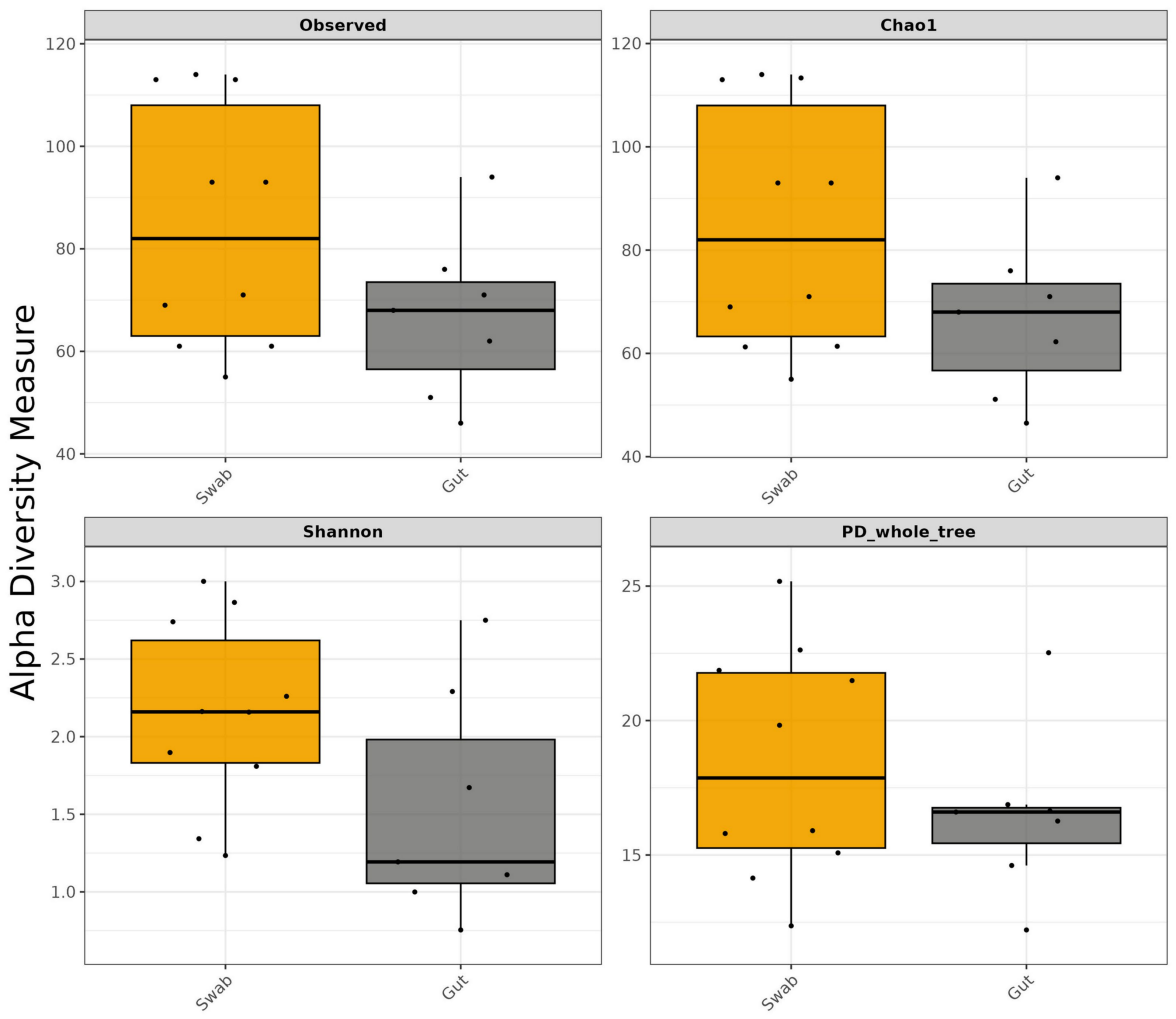
Alpha diversity metrics (Observed, Chao 1, Shannon, and PD_whole_tree) of *B. splendens* intestine microbiota measured using gut and swab sample collection methods. The thick horizontal line of each box represents the median value, while the lower and upper limits of the box are the 25 and 75% quartile, respectively. The horizontal line above and below the box represents the minimum and maximum values of the data, excluding any outliers. These outliers, if present, are depicted as individual points beyond the whiskers.

Alpha diversity metrics were used to evaluate microbial community complexity in the gut microbiota of *B. splendens*, including Observed ASVs, Shannon diversity index, Chao1 richness estimator, and Faith’s PD whole tree index. These parameters quantify taxonomy abundance, community evenness and richness, and evolutionary diversity, respectively. The sequence depth ranged from 44,171 to 53,711 reads across all samples. To standardize sampling effort and mitigate potential bias from sequencing depth variation, rarefaction analysis was performed with a threshold of 42,960 reads per sample (Supplementary Figure 1). Although swab sampling targeted the middle of the intestine by inserting a swab through the mouth of the fish when the intestine sampling targeted the whole intestine, a comparative analysis of alpha diversity metrics showed no statistically significant differences between sampling methods (*p* > 0.05) (Figure 1). However, non-lethal swab samples consistently exhibited higher diversity values across measured indices compared to conventional gut scraping samples (Figure 1).

**Figure 2:**
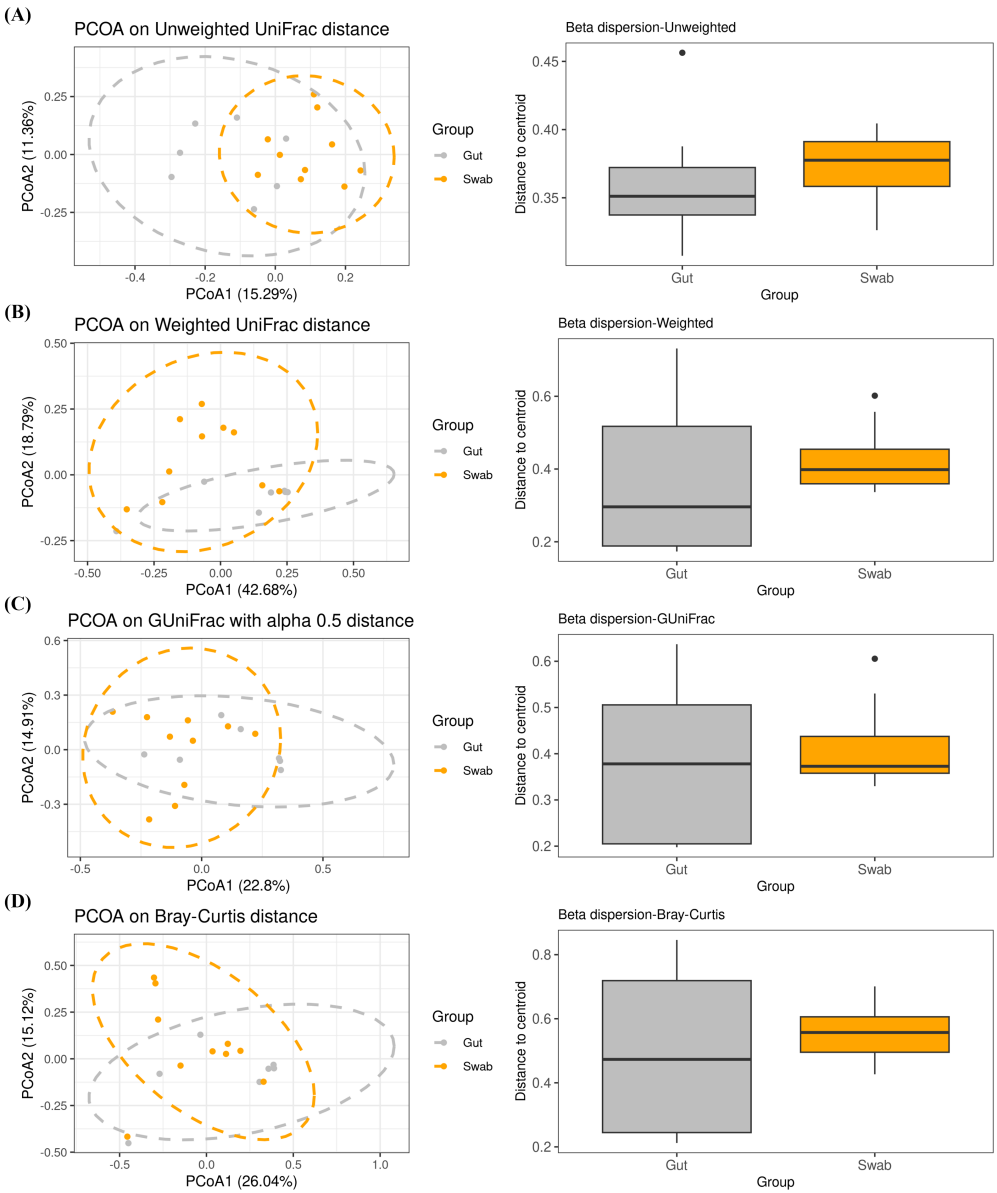
PCoA based on beta diversity showing the spatial distribution of *Betta splendens* gut bacteria using mouth swab and intestine microbiota collection methods **(A–D)**. Unweighted UniFrac **(A)**, weighted UniFrac **(B)**, GUniFrac *α* = 0.5 **(C)**, and **(D)** Bray–Curtis.

Beta diversity was assessed to evaluate compositional dissimilarity between microbial communities across sampling methods. Principal coordinates analysis (PCoA) based on unweighted UniFrac analysis, which emphasizes rare taxa by evaluating presence/absence patterns in a phylogenetic context, demonstrated a significant difference between sampling methods (PERMANOVA, df = 1, *R*^2^ = 0.125, *F* = 2.147, *p* < 0.001; Figure 2A). Similarly, weighted UniFrac analysis, which accounts for both phylogenetic relationships and the relative abundance of taxa, also revealed significant differences between sampling methods (PERMANOVA, df = 1, *R*^2^ = 0.174, *F* = 3.167, *p* = 0.034; Figure 2B). Generalized UniFrac (GUniFrac) with an alpha parameter of 0.5, which adjusts for the influence of high- and low-abundance species while considering their abundance and phylogenetic relationships, further confirmed a significant difference between methods (PERMANOVA, df = 1, *R*^2^ = 0.143, *F* = 2.511, *p* = 0.011; Figure 2C). In contrast, Bray–Curtis dissimilarity metrics revealed no statistically significant differences between sampling groups (PERMANOVA, df = 1, *R*^2^ = 0.077, *F* = 1.252, *p* = 0.220; Figure 2D).

**Figure 3:**
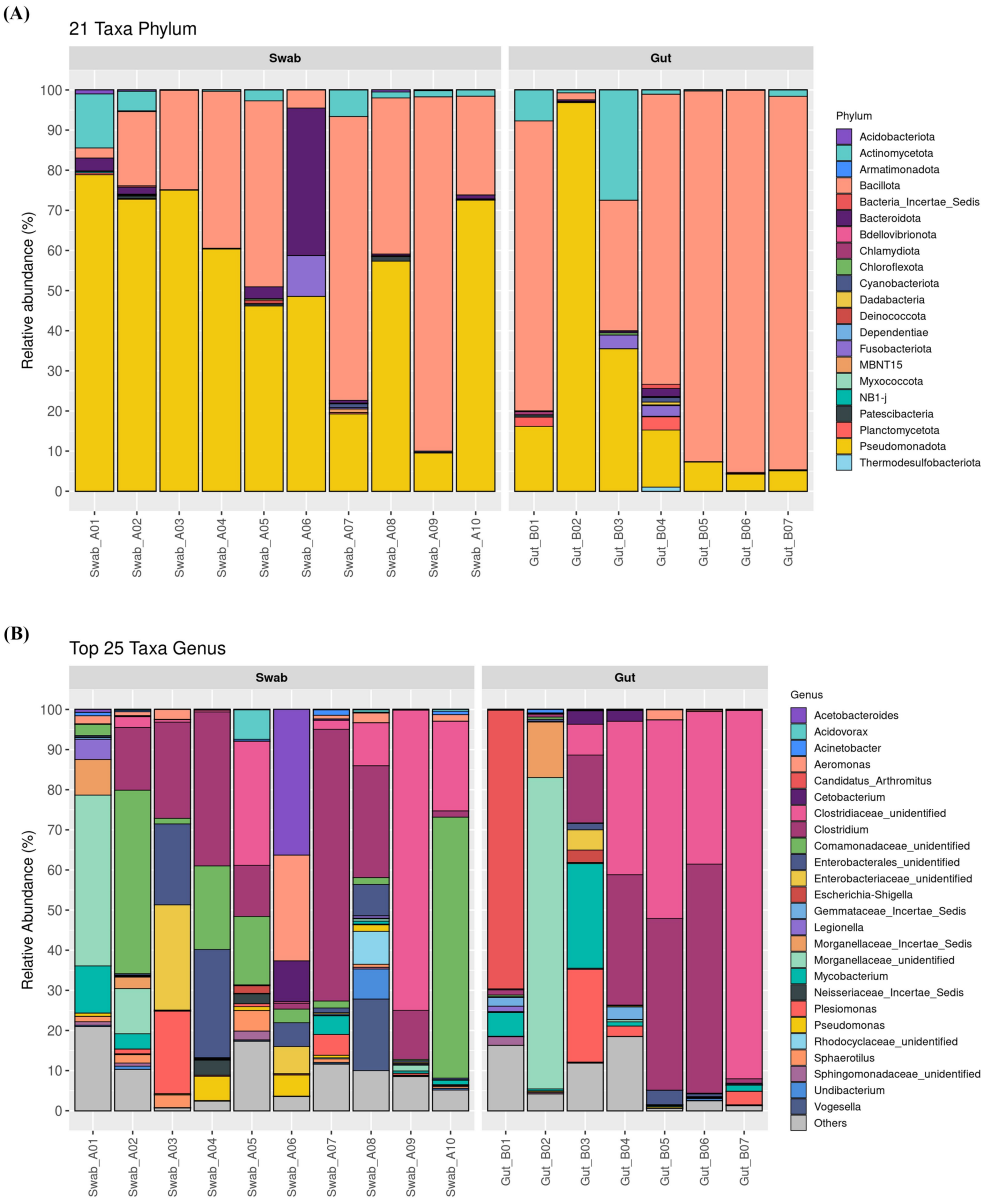
The top abundant microbiota **(A)** phylum and **(B)** genus in *B. splendens* gut obtained with the mouth swab and intestine collection methods.

Quantitative taxonomic analysis revealed differences in bacterial taxon composition profiles between sampling methods at multiple taxonomic levels (Figure 3A). At the phylum level, swab samples exhibited a higher taxonomic diversity with Pseudomonadota (relative abundances: 54.04 ± 7.51%) representing the predominant phylum, followed by Bacillota (35.83 ± 8.66%), Bacteroidota (4.70 ± 3.58%), and Actinomycetota (3.28 ± 1.32%). In contrast, gut samples displayed a less diverse profile dominated by Bacillota (65.64 ± 13.45%), followed by Pseudomonadota (25.61 ± 12.54%), and Actinomycetota (5.56 ± 3.79%). Statistical analysis identified significantly higher relative abundances of Acidobacteria (Wilcoxon, *p* = 0.019) and Pseudomonadota (Wilcoxon, *p* = 0.043) in swab samples compared to gut samples.

An analysis at the genus level revealed the 25 most abundant bacterial taxa (Figure 3B). In swab samples, the most prevalent genera were *Clostridium* (20.17 ± 6.59%), and genera of the families Comamonadaceae (15.99 ± 7.08%), Clostridiaceae_unidentified (14.53 ± 7.51%), and Enterobacteriaceae (3.40 ± 2.62%). In contrast, gut samples were dominated by genera of Clostridiaceae_unidentified (32.22 ± 12.51%), *Clostridium* (21.77% ± 8.62%), genera of the family Morganellaceae (11.26 ± 11.06%) and “*Candidatus arthromitus*” (9.99 ± 9.91%). Among the top 25 genera, genera of the family Comamonadaceae (Wilcoxon, *p* < 0.001), *Pseudomonas* (Wilcoxon, *p* < 0.025), and *Sphaerotilus* (Wilcoxon, *p* < 0.001) exhibited significantly higher relative abundances in swab samples compared to gut samples. Additional taxonomic details are provided in Supplementary Table 1.

**Figure 4:**
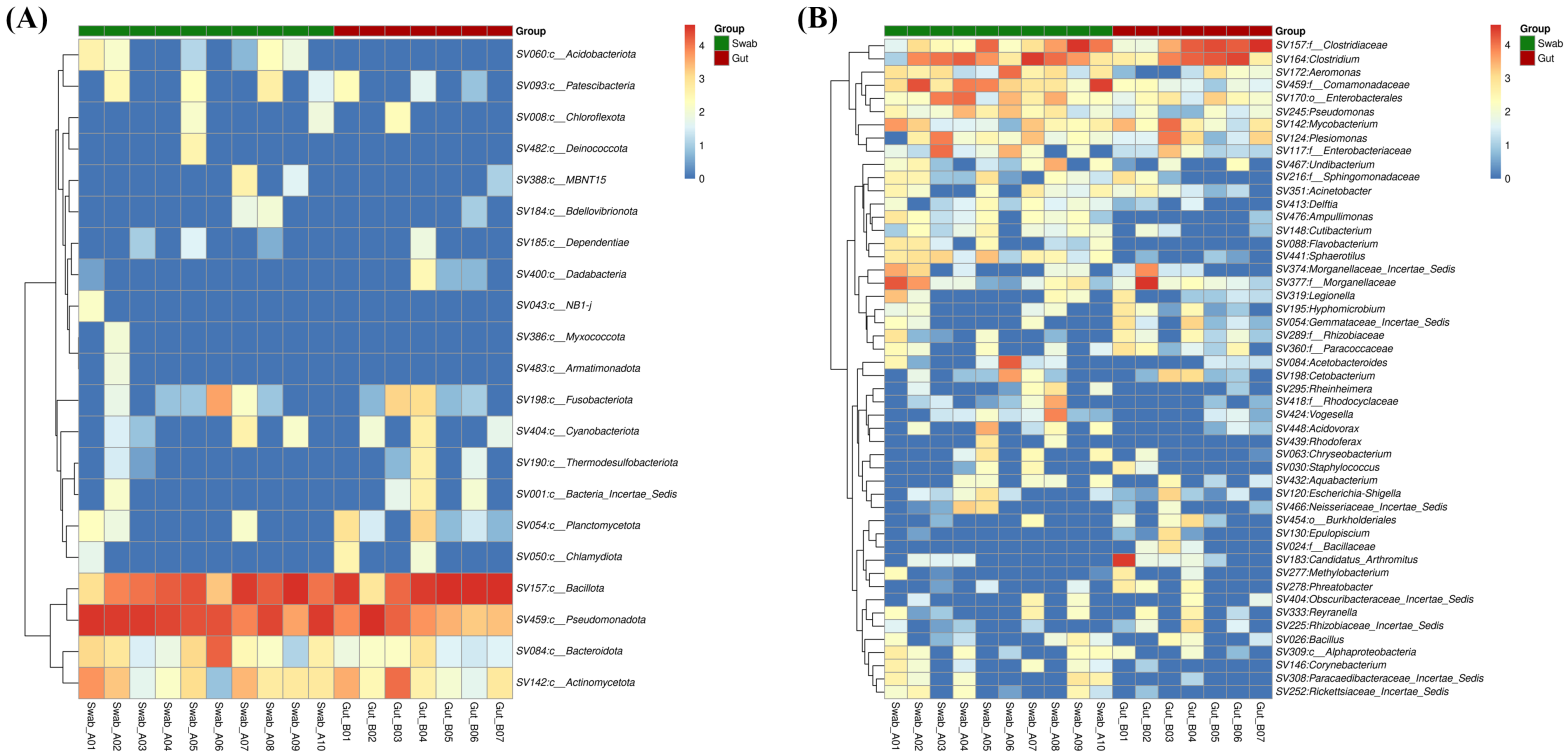
Heatmap hierarchical clustering of *B. splendens* intestine microbiota **(A)** phylum and **(B)** genus using gut and swab collection methods.

Hierarchical clustering analysis with heatmap visualization was performed to elucidate differential abundance patterns of bacterial taxa between sampling methods (Figure 4). At the phylum level, the heatmap demonstrated distinct taxonomic profiles between swab and gut samples (Figure 4A). Four dominant phyla were identified across all samples: Bacillota, Pseudomonadota, Bacteroidota, and Actinomycetota. While Bacillota abundance was higher than mean relative abundance in gut samples (65.64 ± 13.45%) compared to swab samples (35.83 ± 8.66%), this difference did not reach statistical significance (LEfSe, *p* > 0.05).

The heatmap of genus-level presented in Figure 4B, revealed unique patterns of bacterial distribution, with several genera showing preferential detection in swab samples compared to gut samples. This pattern was particularly evident for members of the Pseudomonadota phylum, including Comamonadaceae (unclassified), *Pseudomonas*, and *Sphaerotilus*, which consistently exhibited higher relative abundances in swab samples.

**Figure 5:**
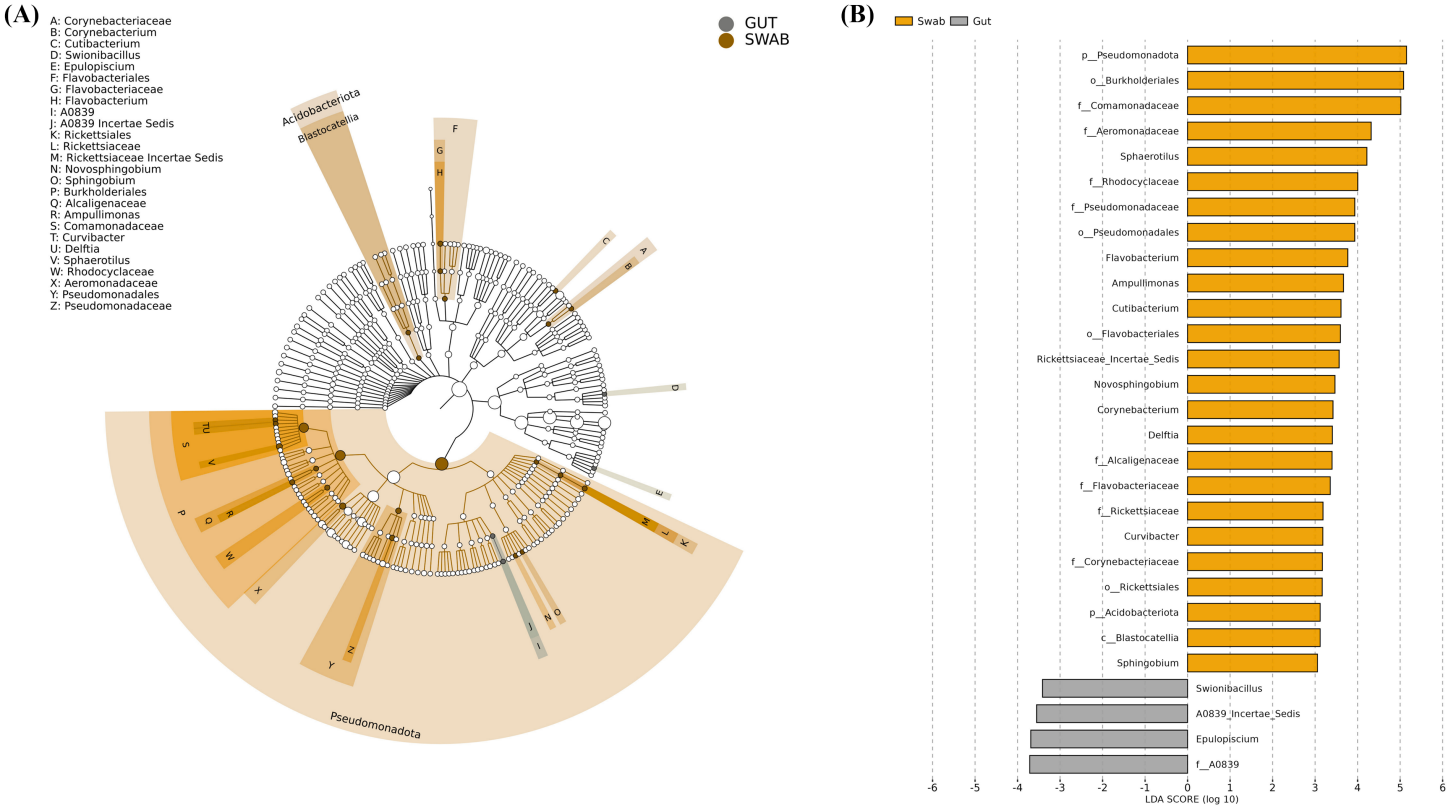
Linear discriminant analysis effect size (LEfSe) of *B. splendens* gut and swab collection method intestine microbiota: **(A)** clustering analysis and **(B)** LDA score.

Linear discriminant analysis effect size (LEfSe) was implemented to identify taxonomic biomarkers distinguishing microbial communities between swab and gut sampling methods (Figure 5). LEfSe results showed that there were significantly different microbial compositions between the swab and gut groups, and some taxa had significant differences corresponding to the sample sources. The swab sample group exhibited enrichment of multiple taxonomic groups, predominantly within the Pseudomonadota phylum. Specifically, the most significantly enriched taxa included at phylum level; Pseudomonadota, order level; Burkholderiales, Pseudomonadales, Flavobacteriales, Rickettsiales, family level; Comamonadaceae, Aeromonadaceae, Pseudomonadaceae, Flavobacteriaceae, genus level; *Flavobacterium*, *Cutibacterium*, *Sphingobium*, etc. In contrast, the gut sample group demonstrates significant enrichment of distinct taxa, including *Swionibacillus*, A0839, and “*Candidatus* Epulopiscium.”

**Figure 6:**
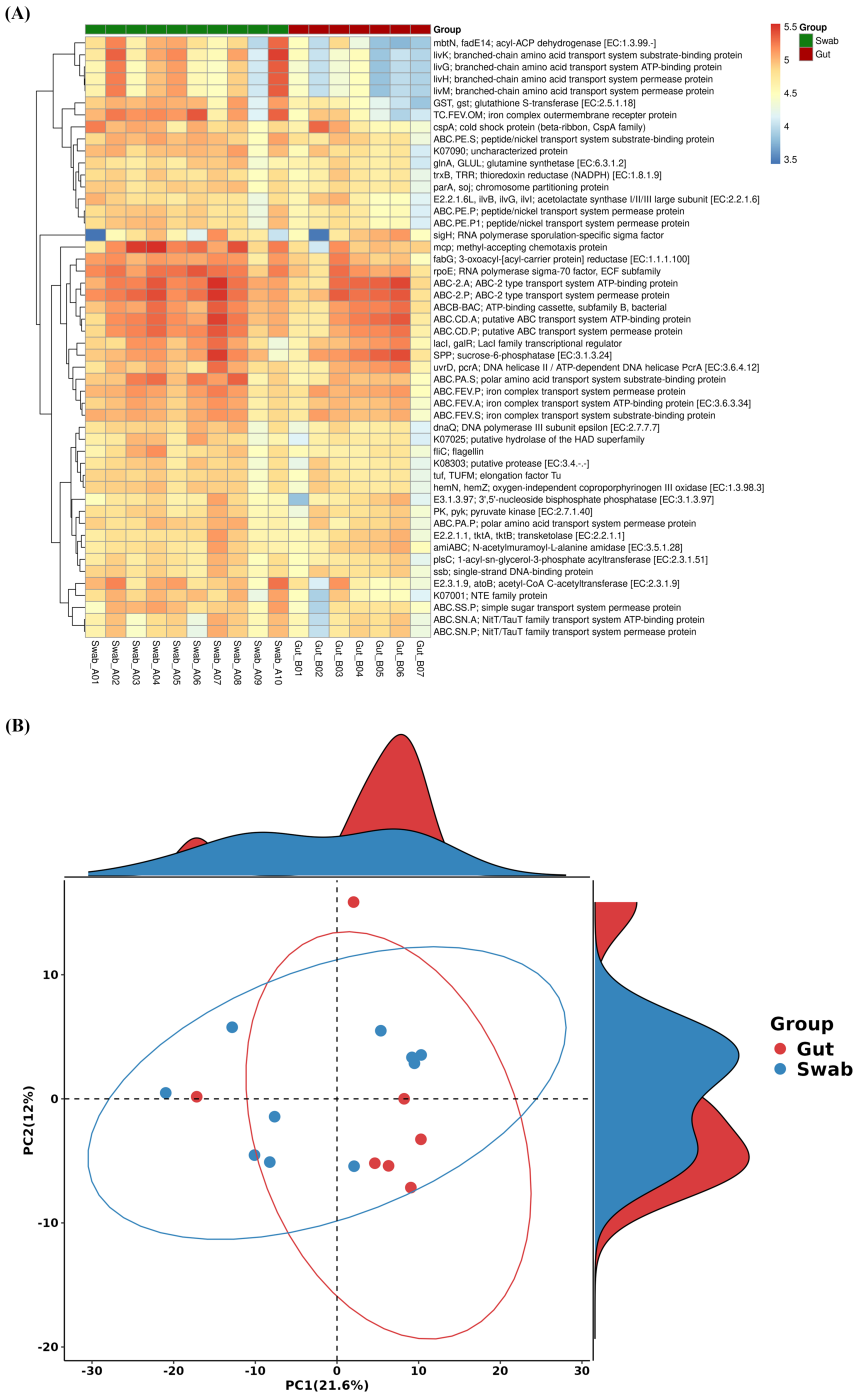
Functional classifications and metabolic pathways based on KEGG (Kyoto Encyclopedia of Genes and Genomes) from *B. splendens* gut microbiota obtained with the swab and intestine collection methods: **(A)** heatmap of orthologous differences and **(B)** PCA of KO function.

Functional potential of the microbial communities was predicted using PICRUSt2 with KEGG pathway analysis to infer metabolic pathways across sampling methods (Figure 6). Principal component analysis (PCA) of predicted functional profiles (Figure 6A) revealed differences in biological functions between samples. The KO heatmap (Figure 6B) illustrates KO function performance across samples, aiding in the study of their metabolic pathways and cellular processes.

Hierarchical clustering of KO functional profiles (Figure 6B) revealed shading differences in predicted metabolic potential. Notably, the swab group showed enhanced gene functions associated with specific metabolic processes, particularly those related to branched-chain amino acids metabolism. Key functional genes demonstrated highlighted activity in swab samples, including mbN (membrane transporter), livK (branched-chain amino acid transporter), livG [ATP-binding cassette (ABC) transporter], liVH (amino acid transporter), and livM (amino acid ABC transporter membrane protein).

Moreover, PCA of predicted functional profiles demonstrated limited separation between sample types, with the first two principal components explaining 33.6% of the total variance (Figure 6B). Although the PCA plot indicates subtle differences in functional profiles between swab and gut samples, the lack of clear cluster separation suggested that the functional potential of microbial communities was largely conserved across the two sampling methods.

## 4 Discussion

As predicted, our results show that gut microbiota diversity obtained by intestine collection and mouth swabbing methods in female *B. splendens* are at least partially comparable in most metrics. Various indexes of alpha diversity, along with the Bray–Curtis dissimilarity index, revealed no significant differences between both methods, indicating that swab and gut samples yield similar richness, evenness, and overall compositional profiles of the gut microbiota. The lack of significant differences in the alpha diversity dissimilarity index between both methods was also found in the rainbow trout *O. mykiss*, and in the striped plateau lizard *Sceloporus virgatus* (9). In contrast, phylogenetic metrics including unweighted UniFrac, weighted UniFrac, and GUniFrac (*α* = 0.5) revealed significant differences between methods. This divergence implies that the sampling method can influence the observed profile, particularly regarding rare species membership and the relative abundance of taxa.

Hierarchical clustering analysis supports these observations, suggesting that swab samples may harbor more phylogenetically diverse taxa, potentially reflecting the specialized microenvironment of upper intestinal mucosa. This effect was also found in *O. mykiss* (14), using Bray–Curtis and weighted UniFrac measures, with swab samples forming clear phylogenetic clusters.

These results are also supported by data from other taxonomical groups. In cane toads (*Rhinella marina*) cloacal swabs effectively captured the large intestine microbiota but differed significantly from small intestine communities (12). In ostriches (*Struthio camelus*), Videvall et al. (11) found that all beta diversity metrics showed differences, with swab samples displaying more dispersed clustering compared to the tighter clustering of gut samples. Likewise, in striped plateau lizards (*Sceloporus virgatus*), notable differences were observed between weighted UniFrac values of swab and gut microbiota (9). The gut microbiota exhibits more pronounced structural and compositional differences between populations than within a single sample and in a previous study the use of swab sampling in fish also suggested similar results (14, 32). However, the taxonomic analysis shows that non-lethal swabbing identified the majority of the microbial groups also identified by lethal gut sampling, plus some additional groups only present when sampled by the swab method. These results suggest that, while the two sampling methods capture distinct phylogenetic profiles, they yield equivalent representations of the predominant gut microbiota at a broad taxonomic level relevant to many ecological comparisons. Differences in beta diversity and taxonomic composition may be attributable to underlying variation in phylogenetic structure or to differences in rare species composition. Thus, from the perspective of overall community phylogeny, detectable and significant differences exist between gut content and oral swab sample groups, whereas dominant microbial membership remains consistent. This interpretation is further supported by principal component analysis of predicted functional profiles. Swab sampling is a valuable tool for monitoring intervention experiments on *B. splendens* as well as conservation efforts for small fish, given that this method does not require euthanizing the individuals being sampled. Note that swab sampling involves passing the swab through the upper part of the digestive tract, including the oral cavity, leading to the collection of bacterial groups present in these regions but absent in the intestine. While the oral cavity is rich in nutrients, prior to their digestion, and includes a mix of aerobic, micro-aerophilic and anaerobic niches; the intestinal section of the digestive tract is predominantly anaerobic (33) and is at the end of the digestive process resulting in more restricted and simpler set of nutrient types. These microenvironmental conditions would lead to a shift in the native microbial communities (34), which is reflected in the alpha and beta diversity patterns detected within our samples.

Taxonomic analysis revealed distinctive microbial profiles between sampling methods. Swab samples exhibited a higher abundance of bacteria from the phylum Pseudomonadota (formerly Proteobacteria), identified as a predominant phylum in fish intestinal microbiota in previous studies (35, 36). Pseudomonadota represents a diverse group of bacteria with related roles in nutritional metabolism, immune regulation, and disease resistance function (37, 38). Many species of Pseudomonadota are aerobic and associated with biofilms (39), so the increase in abundance of this group might be related to the aerobic conditions of the oral cavity that would promote their growth, as well as with possible foraging of biofilms in exposed substrates of the aquaria. Swab samples also revealed a significantly higher abundance of bacteria from the phylum Acidobacteria, a bacterial group known for their role in carbohydrate metabolism (40), and their preference for colonizing acidic microenvironments (41, 42). Research in this phylum is limited, but studies show that Acidobacteria are more abundant in the gut of filter-feeding fish than in carnivorous fish (43). In contrast, although not statistically significant, gut samples exhibited elevated proportions of Bacillota (formerly Firmicutes). This phyla has been previously reported as dominant members in the gut microbiota of herbivorous fish, possessing specialized enzymatic activities for complex carbohydrate metabolism (44, 45). A previous gut microbiota study on *B. splendens* by Gruneck et al. (17) also indicates that Pseudomonadota and Bacillota constituted the predominant bacterial phyla, which is consistent with our results.

The taxonomic distinction observed between sampling methods may indicate functional specialization within the gut microbiome. At genus level, members of the family Clostridiaceae, including Clostridiaceae_unidentified, *Clostridium* and *Candidatus_Arthromitus*, most observed in the gut samples, are known for their roles in fermenting dietary fiber (DF) and producing short-chain fatty acids (SCFAs) (43, 46). SCFAs play a key role in modulating anti-inflammatory responses in epithelial cells (47, 48), and facilitating gut–brain communication (49). Comamonadaceae may have a potential association with lipid metabolism, feeding bacterial protein feed containing Comamonadaceae, a decrease in fat deposits was observed (50), moreover, the proliferation of Comamonadaceae may be related to the anti-inflammatory state of the intestine (51). *Enterobacteriaceae*, as a group of facultative anaerobes, contribute to maintaining an effective anaerobic environment in the intestine, vitamin K biosynthesis, and preventing intestinal pathogen infection (52), with some genera also contributing to SCFA production (53). Members of Morganellaceae exhibit antagonistic activity against pathogenic microbes (54, 55). Meanwhile, significant differences in the relative abundance of Comamonadaceae_unidentified, *Pseudomonas*, *Sphaerotilus* between sampling methods, along with hierarchical clustering results, suggest that Pseudomonadota may exhibit higher phylogenetic richness in the gut microbiota of *B. splendens*. *Pseudomonas* is an opportunistic pathogen but also has been linked to protein digestion, nutrient absorption, and antimicrobial metabolite production (56–59). The increased BCAA-related functional pathway observed in swab samples exhibiting stronger gene functions associated with branched-chain amino acid (BCAA) metabolism are also likely due to a higher abundance of *Pseudomonas*. *Sphaerotilus* (Pseudomonadota) may related to cellulose degradation ability (60). In this study, the higher abundance of Comamonadaceae_unclassified in swab samples aligns with previous findings indicating enrichment in mucosa-associated microbiota (61).

Gruneck et al. (17) found that *Aeromonas* and *Plesiomonas* (Pseudomonadota) were the most predominant genus of gut microbiota in adult *B. splendens* while we identified *Clostridium* (Bacillota), Comamonadaceae_unidentified (Pseudomonadota), and Clostridiaceae_unclassified (Bacillota) (Supplementary Table 1). That difference may be due to factors such as individual fish genetic background (62), environmental conditions such as temperature (63), dietary habits (64), cultural environment (65, 66), or salinity (67).

LEfSe identified distinct taxonomic profiles between sampling methods. Many genus found in the swab samples are likely associated with bacteria living in the oral cavity, such as *Flavobacterium* that are known for their preference for aerobic conditions (68), and are associated with diseases that affect mostly the oral cavity and gills. This genus is particularly predominant in fish grown in artificial settings [e.g., (68, 69)]. Similarly, *Sphingobium* is a genus also associated with aerobic conditions and very common in both soil and water samples (70, 71), and previous studies have detected members of this genus in gill and stomach mucosae of fish but not in their intestines (72). From bacteria found in the gut, the most notable is “*Candidatus* Epulopiscium” that represents a genus of giant symbiotic bacteria largely found in surgeon fish (*Acanthuridae leucosternon*) living in coral reefs across different regions (73), and it was the first time being reported in *B. splendens*. If the presence of this group is confirmed, it would significantly extend the ecological range of “*Candidatus* Epulopiscium.” This genus was found mostly in the gut samples, and it suggests that while swab sampling technique effectively captures mucosal-associated microbiota, it may underrepresented bacteria residing within the epithelium and/or in the basal region of intestinal villi. This pattern aligns with findings from fish gill microbiome studies, indicating that swab sampling enhances the richness and diversity of the microbiome compared to biopsy (32), and supports previous research on the regulatory role of the intestinal epithelium in shaping microbial communities (74). However, it is also worth noting that these giant bacteria are known to have a daily cycle, with movement from the lower to the upper part of the intestine, reflecting feeding patterns of their hosts and nutrient availability (75). Swabbing results might be a reflection of sampling timing coinciding with a period where members of this microbial group are located in a more distal part of the intestinal tract, which is less accessible with this technique. Alternatively, the significant dimension of their cells might also be hampering their successful collection using swabs.

Overall, we find that swab sampling is an effective method for studying *B. splendens* gut microbiota, but we also caution that this type of sampling also captures microbiota residing in the upper part of the digestive system. Therefore, the selection of an appropriate sampling method should be guided by specific research objectives in future studies. Non-lethal sampling can reduce disturbance to fish populations, benefit animal welfare, and provide the opportunity to study changes in the microbial communities of individual fish (7). Furthermore, the non-lethal swab technique could be employed in longitudinal studies to investigate the influence of different developmental stages, dietary interventions, and social or ecological factors on the gut microbiome dynamics within individual organisms. However, although swab collection is a non-lethal method, repeated sampling in long-term studies may still cause some stress to the fish, which may affect the microbiota composition, so it should be used with caution and further studies are needed to assess its effect on fish. Nonetheless, this method has potential, especially under multiple sampling, to understand the changes of *B. splendens* gut microbiome over time. Given the status of *B. splendens* as a commonly used model for the study of aggression, the swab technique could present itself as an opportunity to study how gut microbiota might affect this behavior and vice-versa, in a longitudinal way, for example by analyzing how the microbial community can differ when individuals are subjected to different social context, or by studying the ontogenetic changes during the development of individuals. It should be noted that the conclusions of this study are primarily derived from observations and experiments on female *B. splendens* and, in this species, there is significant differences in male and female aggressive behavior. However, observations from dissected fish in our lab suggest that the digestive tract of male and female fighting fish are similar. Oral swab sampling should thus be equally applicable to both sexes. In other fish species, given the limited number of studies utilizing swab sampling, the broader applicability of this method remains to be established. Future research should aim to include a broader range of fish species and assess individuals raised in diverse environmental conditions.

## Data Availability

The nucleic acid sequences in this study are available in the NCBI Sequence Read Archive database: PRJNA1321277.
