# Supplementary material for: A non-lethal method for the sampling of the gut microbiota in Betta splendens
Source: Front Vet Sci. 2026 Feb 5;13:1702535. doi: 10.3389/fvets.2026.1702535 (PMC12917903; doi:10.3389/fvets.2026.1702535)
Supplement: Supplementary file 2 [file Presentation_1.pdf]

Supplementary Material

1.1 Supplementary Table 1

1.2 Supplementary Figure 1

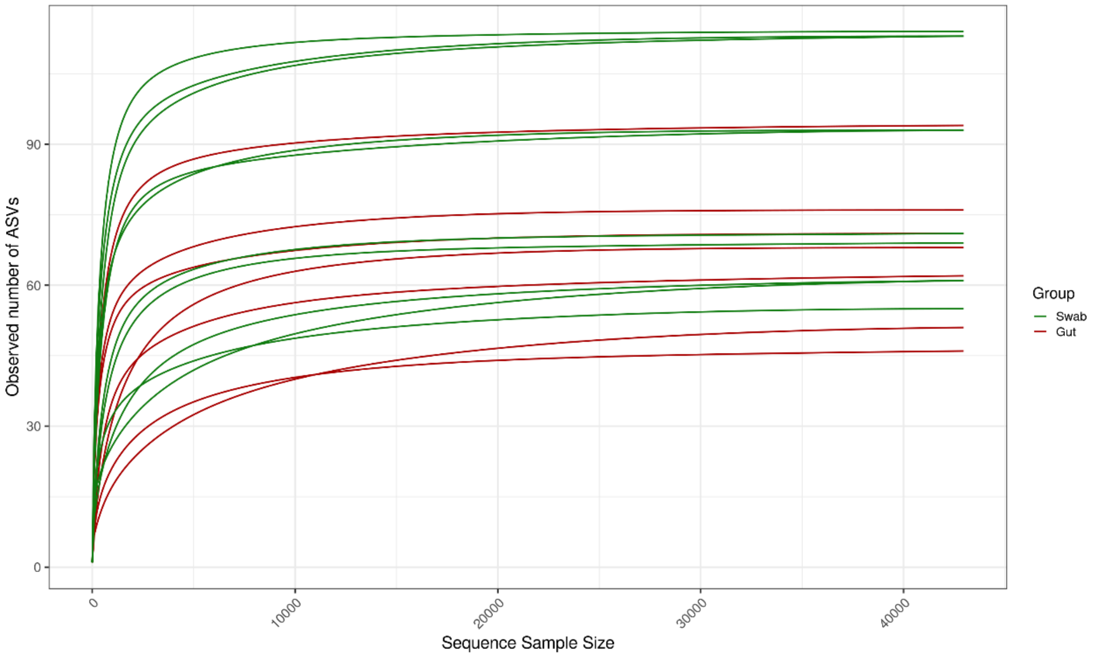

Supplementary Figure 1. The rarefaction analysis to standardize sampling effort. The x-axis represents the sequence sample size (number of reads) and the y-axis shows the observed number of Amplicon Sequence Variants (ASVs). The curves indicate observed ASVs increase with sample size, highlighting the impact of sequencing depth. The analysis used a threshold of 42,960 reads per sample.
